# Treatment outcomes in patients with pyogenic vertebral osteomyelitis who have cirrhosis

**DOI:** 10.1038/s41598-019-51758-w

**Published:** 2019-10-23

**Authors:** Jihye Kim, Ho Suk Kang, Jeoung Woo Kim, Seok Woo Kim, Jae-Keun Oh, Young-Woo Kim, Moon Soo Park, Tae-Hwan Kim

**Affiliations:** 1 Division of Infection, Department of Pediatrics, Kangdong Sacred Heart Hospital, Hallym University College of Medicine, Seoul, Korea; 2 Division of Gastroenterology and hepatology, Department of Internal Medicine, Hallym Sacred Heart Hospital, Hallym University College of Medicine, Anyang, Korea; 3 Spine Center, Department of Orthopedics, Hallym University Sacred Heart Hospital, Hallym University College of Medicine, Anyang, Korea; 4 Department of Orthopaedic Surgery, Hallym University Dongtan Sacred Heart Hospital, Hallym University College of Medicine, Hwasungsi, South Korea

**Keywords:** Bacterial infection, Risk factors

## Abstract

Early diagnosis and proper treatment of pyogenic vertebral osteomyelitis (PVO) in patients with cirrhosis is challenging to clinicians, and the mortality rate is expected to be high. A retrospective study was conducted to investigate the treatment outcome in PVO patients with cirrhosis and to identify the predictors of their mortality. Mortality was divided into two categories, 30-day and 90-day mortality. A stepwise multivariate logistic regression model was used to identify predictors of mortality. Eighty-five patients were identified after initial exclusion. The patients’ mean age was 60.5 years, and 50 patients were male. The early mortality rates within 30 and 90 days were 17.6% and 36.5%, respectively. Multivariate analysis revealed that increased age, CTP class C, and bacteremia at the time of PVO diagnosis were predictors of 30-day mortality, while higher MELD score, presence of combined infection, and multiple spinal lesions were predictors of 90-day mortality. Attention should be paid to the high mortality between 30 and 90 days after PVO diagnosis (18.8%), which was higher than the 30-day mortality. Liver function was consistently a strong predictor of mortality in PVO patients with cirrhosis. The high-risk patients should be targeted for an aggressive diagnostic approach, using spinal MRI and intensive monitoring and treatment strategies.

## Introduction

The mortality in liver cirrhosis was reported to be greater than that in the five major cancers^1^. Infection further increases the mortality of patients with cirrhosis by fourfold^2^, and infection is directly responsible for 30–50% of deaths in patients with cirrhosis^3,4^. Considering the greatly increased mortality from infection in patients with cirrhosis^2^, early diagnosis and prompt treatments should be compulsory to save patients’ lives. However, adherence to such a basic principle for patients with cirrhosis is not easy for clinicians engaged in the treatment of pyogenic vertebral osteomyelitis (PVO).

A retrospective study reported that 77% of patients with end-stage liver disease had bodily pain, in the abdomen, back, head/neck, and upper and lower extremities, within 24 hours of the evaluation, and most patients (90%) received various analgesics^5^. Such a high prevalence of bodily pain in patients with cirrhosis prevents the use of the clinical symptoms of PVO as an indicator for early diagnosis. In addition, bacterial infections are common in immunocompromised patients with cirrhosis^6^, and patients with cirrhosis have a fivefold greater risk of developing infection than the general population^7^. Such high prevalence of bacterial infection in patients with cirrhosis also prevents the use of infection markers as indicators for early diagnosis of PVO. According to the guideline of the Infectious Diseases Society of America, spine magnetic resonance imaging is recommended for patients with suspected PVO who have new or worsening back pain and elevated erythrocyte sedimentation rate or C-reactive protein level^8^. However, such an approach is considered to have limitations in PVO patients with cirrhosis.

Treatment of PVO in patients with cirrhosis is challenging for clinicians. Attenuated liver function by bacterial infection threatens the life of patients with cirrhosis through variceal rupture^9^ and multiorgan failure^10^. Treatment failure or recurrence is expected to be high, owing to cirrhosis-associated immune dysfunction^11^ and the high prevalence of multidrug resistant organisms^12^. The strictly required long-term intravenous antibiotics^8^ to reduce the recurrence of PVO potentially can paradoxically cause *Clostridium difficile* infection, which is associated with higher mortality^13^. Decreased bone mineral density with deteriorated bony microarchitecture in patients with cirrhosis^14^, disuse osteoporosis caused by immobilization, and long-term hospitalization aggravates skeletal destruction by the pyogenic organism, and can easily cause neurological and structural instabilities that require surgical treatment. However, the basic principles in the surgical treatment of PVO^15^, including sufficient removal of paraspinal abscesses and firm spinal instrumentation, are technically challenging in patients with cirrhosis who have poor bone quality and bleeding tendency with coagulopathy.

As a result, difficulty in early diagnosis and prompt treatments in PVO patients with cirrhosis is expected to be related to poorer clinical outcomes, including higher mortality. However, to our knowledge, no reports have described the treatment outcome in this patient group. In addition, under the expected higher mortality, prognostic studies to identify high-risk patients, on whom intensive monitoring and treatment strategies should be concentrated, are essential for the improvement of clinical outcome. We performed a retrospective study to investigate the treatment outcome in PVO patients with cirrhosis, and to identify the predictors of their mortality.

## Methods

### Study design and ethics

A retrospective case review was performed in patients with cirrhosis who received treatment for PVO in our institution between January 2000 and March 2018. This study was designed and conducted using the format recommended by STROBE (Strengthening the Reporting of Observational Studies in Epidemiology) guidelines^16^. This study was approved by the institutional review board of Hallym University Sacred Heart Hospital. The institutional review board waived the informed consent for this study. All methods were carried out in accordance with the relevant guidelines and regulations.

### Study patients

Our university medical center is one of the largest medical institutions in our country, consisting of six general hospitals. This study was performed in the main institute among the six general hospitals. As the main institute of our medical center, our hospital serves as a tertiary referral center for not only the other five general hospitals but also numerous local hospitals.

Patients with cirrhosis who received treatment for PVO were eligible for inclusion. Cirrhosis was diagnosed by liver pathological examination or a combination of laboratory biochemical, radiological, and endoscopic findings, if a liver biopsy result was not available^14^. PVO was defined using the following criteria: suggestive clinical symptoms, accompanying typical radiological features on MRI, and microbiological identification^15^. Microbiological confirmation included isolation from blood culture, CT-guided needle biopsy, or surgical biopsy. Patients were excluded if their medical records indicated that they had received a previous liver transplantation before the diagnosis of PVO. Patients were also excluded if they had received a previous spine surgery using instrumentation at the same site of the PVO. Other reasons for exclusion were incomplete medical records or imaging data.

### Data collection

Data were retrieved from electronic medical records using a standardized collection form. Demographic, laboratory, and other clinical data at the time of PVO diagnosis were ascertained. Medical history was retrieved from the records, and the Charlson comorbidity index was calculated to assess comorbid medical conditions^17^. The presence of ascites, encephalopathy, and gastrointestinal (GI) bleeding at the time of PVO diagnosis were retrieved from the records, and laboratory data at the time of PVO diagnosis were retrieved. Then, liver function was determined using the Model for End-Stage Liver Disease (MELD), Child-Turcotte-Pugh (CTP) class, and CTP scores. The severity of infection at the time of PVO diagnosis was retrieved using a validated classification system by Pola *et al*.^18^, which divided pyogenic spondylodiscitis into three types as follows: 1) type A, cases without biomechanical instability, neither acute neurological impairment nor epidural abscesses; 2) type B, cases with radiological evidence of significant bone destruction or biomechanical instability without acute neurological impairment or epidural abscesses; and 3) type C, cases with epidural abscesses or acute neurological impairment.

### Definitions

The presence of combined infection was retrospectively retrieved from the medical records and classified as follows:

1. Intra-abdominal infection: Spontaneous bacterial peritonitis was diagnosed on the basis of an ascitic fluid neutrophil count of >250/mm^3^ or a positive bacteriological culture of the ascitic fluid^19^. Infectious enterocolitis was diagnosed in patients with diarrhea and leukocytes in stool or positive stool culture for pathogens, including Salmonella, Shigella, Yersinia, Campylobacter, and pathogenic *Escherichia coli*, or a positive *Clostridium difficile* stool assay^7^.
2. Urinary tract infection: Urinary tract infection included both laboratory-confirmed UTI defined by the presence of pyuria (>10 white blood cells/mm^3^ per high-power field) and bacteria (urinary pathogen of ≥10^5^ colony-forming units per mL)^20,21^, and asymptomatic bacteriuria defined by the presence of 1 or more species of bacteria growing in the urine at specified quantitative counts (≥10^5^ colony-forming units [CFU]/mL or ≥10^8^ CFU/L) irrespective of the presence of pyuria^22^.
3. Cardiac infection: Infective endocarditis was diagnosed using the modified Duke criteria^23^.
4. Pneumonia: At least one of the respiratory symptoms with one of the following: rales and/or crepitation on auscultation; at least one sign of infection in the absence of antibiotics; presence of pulmonary infiltrate on radiological imaging; or positive sputum culture^7^.
5. Other musculoskeletal infections: Septic arthritis was diagnosed on the basis of a synovial fluid leukocyte count of >50,000 cells/μL or positive synovial fluid culture^21^. Osteomyelitis was diagnosed on the basis of typical radiological findings on MRI or positive culture results^24–26^.

Multiple spinal lesions were defined when the spinal involvement presented beyond two vertebral bodies on MRI, with at least one completely uninvolved vertebral body between the involved vertebral bodies. Early surgery was defined as a surgical treatment performed under general anesthesia within 30 days after PVO diagnosis.

### Outcomes

The mortality of the patients was divided into two categories, early and late mortality. Early mortality within 30 or 90 days after PVO diagnosis were investigated. The clinical outcomes were investigated in patients with at least a 90-day survival. Recurrence was defined as having recurrent symptoms and signs after the completion of antibiotics and receiving a second course of intravenous antibiotics^15^.

### Statistical analyses

Continuous variables were presented as the mean ± standard deviation and compared using an independent *t* test. Categorical variables were presented by frequency (%) and compared using the Pearson chi-square test, Fisher exact test, or linear-by-linear association.

Predictors of 30- and 90-day mortality were analyzed using the logistic regression model, and all variables identified as significant in the univariate analysis (p < 0.05) were included in the stepwise multivariate logistic regression model. The Kaplan-Meier survival curve was used to display the cumulative probability of late survival in patients, and the log-rank test was used to compare survival curves between the two groups.

The statistical tests were two-tailed, and a p value of <0.05 was considered to indicate statistical significance. All the analyses were performed using SPSS 24 (SPSS Inc., Chicago, Illinois, USA).

## Results

### Baseline Patient Characteristics

**Figure 1:**
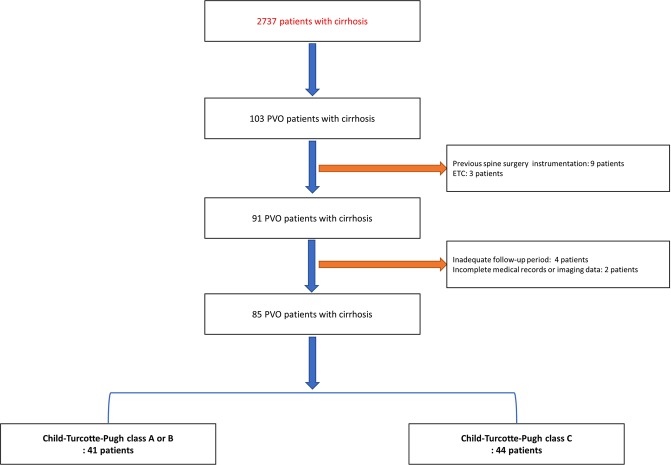
Flowchart of patients included in the study. Abbreviations: PVO, pyogenic vertebral osteomyelitis.

Eighty-five patients were identified after the initial exclusion (Fig. 1). The patients’ mean age was 60.5 years, and 50 patients (58.9%) were male (Table 1). The most common etiology of cirrhosis was viral hepatitis (55.3%). Ascites, encephalopathy, and GI bleeding were present at the time of PVO diagnosis in 28 (32.9%), 20 (23.5%), and 16 patients (18.8%), respectively. Seven, 34, and 44 patients had CTP class A, B, and C cirrhosis, respectively. Hepatocellular carcinoma was identified in 16 patients (18.8%).

**Table 1:** Baseline patient characteristics.

Variables	Categories of variables	All patients	Child-Turcotte-Pugh class A or B	Child-Turcotte-Pugh class C	p-value
Number of patients		85	41	44	
Age		60.5 ± 8.7	58.7 ± 8.6	62.1 ± 8.6	0.068
Sex ratio (F: M)		35: 50	18: 23	17: 27	0.622
BMI (kg/m^2^)		24.4 ± 2.9	24.2 ± 2.9	24.5 ± 3.0	0.574
Etiology of cirrhosis	Viral	47 (55.3)	19	28	0.289
	Alcoholic	29 (34.1)	18	11	
	others	9 (10.6)	4	5	
Charlson comorbidity index score		5.8 ± 3.2	5.1 ± 2.8	6.4 ± 3.4	0.066
Medical history	Coronary artery disease	10 (11.8)	4	6	0.740
	End stage renal disease	12 (14.1)	3	9	0.120
	Diabetes Mellitus	41 (48.2)	12	29	0.002
	Overall malignancy	23 (27.1)	11	12	0.963
	Hepatocellular carcinoma	16 (18.8)	4	12	0.053
Child-Turcotte-Pugh class (A/B/C)		7/34/44	7/34/0	0/0/44	
Child-Turcotte-Pugh score		9.6 ± 2.5	7.3 ± 1.0	11.8 ± 1.3	<0.001
MELD score		23.0 ± 12.1	12.9 ± 5.4	32.3 ± 8.4	<0.001
Morbidity related with cirrhosis	Ascites	28 (32.9)	0	28	<0.001
	Encephalopathy	20 (23.5)	0	20	<0.001
	GI bleeding	16 (18.8)	0	16	<0.001
Laboratory data	WBC (×10^3^/μL)	9.8 ± 3.9	9.4 ± 3.7	10.1 ± 4.1	0.424
	Platelet (×10^3^/μL)	10.4 ± 7.1	138 ± 82	72 ± 37	<0.001
	Serum albumin (g/dl)	2.4 ± 0.6	2.5 ± 0.6	2.4 ± 0.6	0.400
	Total bilirubin (mg/dl)	6.3 ± 5.3	2.5 ± 2.0	9.9 ± 5.0	<0.001
	Prothrombin time (INR)	2.1 ± 1.1	1.2 ± 0.3	2.9 ± 0.9	<0.001
	Serum creatinine (mg/dl)	2.0 ± 1.7	1.4 ± 1.2	2.5 ± 2.0	0.003
	C-reactive protein (CRP, mg/L)	75 ± 35	79 ± 38	70 ± 32	0.214
	Erythrocyte sedimentation rate (ESR, mm/h)	60 ± 27	62 ± 30	59 ± 26	0.627
Neurologic deficit by ASIA grade	A	0	0	0	0.906
	B	5 (5.9)	3	2	
	C	12 (14.1)	7	5	
	D	43 (50.6)	17	26	
	E	25 (29.4)	14	11	
Bacteremia		55 (64.7)	23	32	0.109
Combined infection	Presence of combined infection	48 (56.5)	18	30	0.024
	Intraabdominal	7 (8.2)	1	6	0.110
	Urinary tract	26 (30.6)	10	16	0.231
	Cardiac	4 (4.7)	3	1	0.349
	Pneumonia	19 (22.4)	5	14	0.038
	Other musculoskeletal	12 (14.1)	3	9	0.120
	Others	8 (9.4)	4	4	1.000
Spinal anatomical involvement	Single	56 (65.9)	30	26	0.171
	mainly cervical	4 (4.7)	3	1	
	mainly thoracic	13 (15.3)	10	3	
	mainly lumbosacrum	39 (45.9)	17	22	
	Multiple	29 (34.1)	11	18	
Number of infected vertebral bodies	within 3 levels	33 (38.3)	23	10	0.002
	over 3 levels	52 (61.2)	18	34	
Severity of infection by Pola *et al*.	Type A	5 (5.9)	5	0	0.031
	Type B	8 (9.4)	4	4	
	Type C	72 (84.7)	32	40	
Causative organism of PVO	Staphylococcus aureus	42 (49.4)	19	23	0.560
	Methicillin resistant	21 (24.7)	9	12	
	Methicillin sensitive	21 (24.7)	10	11	
	Other gram positive bacteria	15 (17.6)	7	8	
	Enterobacteriaceae	18 (21.2)	10	8	
	Others	10 (11.8)	5	5	

Data were presented by number (%) of patients or mean ± standard deviation.

Bacteremia was present in 55 patients (64.7%; Table 1). Combined infection was present in 48 patients (56.5%), and urinary tract infection was the most common (26 patients, 30.6%). Most of the patients (56 patients, 65.9%) had a single spinal lesion, but multiple spinal lesions were observed in 29 patients (34.1%). The number of infected vertebral bodies was >3 levels in 52 patients (61.2%), and most patients had type C infection according to the classification of Pola *et al*. (72 patients, 84.7%). The most common causative organism was *Staphylococcus aureus* (42 patients, 49.4%), and it was methicillin resistant in half of the patients (21/42 patients).

CTP class C patients had a significantly increased number of infected vertebral bodies (p = 0.002) and severe types of infection according to the classification of Pola *et al*. (p = 0.031) when compared to the CTP class A or B patients (Table 1). Combined infection was more frequent in the CTP class C patients (p = 0.024).

Surgical treatment was performed in 10.6% (9 of 85 patients) within one week of PVO diagnosis, and 29.4% between one and four weeks after PVO diagnosis (25 of 85 patients).

### Mortality of PVO patients with cirrhosis

**Figure 2:**
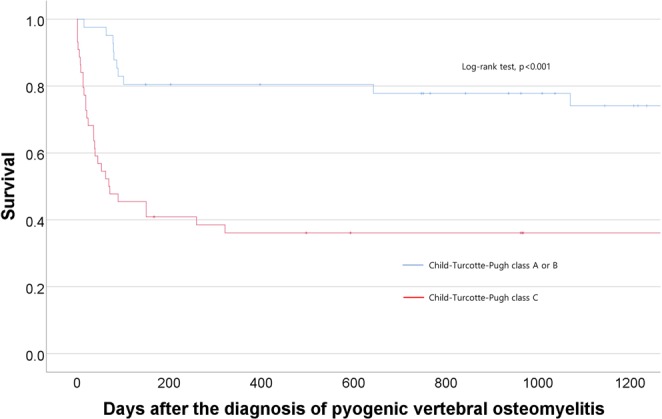
Cumulative probability of survival for PVO patients with cirrhosis according to Child-Turcotte-Pugh class.

The early mortality rates within 30 and 90 days were 17.6% (15/85 patients) and 36.5% (31/85 patients), respectively (Table 2). The CTP class C patients had greatly increased 30-day (31.8% vs 2.4%, p < 0.001) and 90-day mortality (54.5% vs 17.1%, p < 0.001) when compared to the CTP class A or B patients (Fig. 2).

**Table 2:** Mortality of PVO patients with cirrhosis.

Categories of mortality		All patients	Child-Turcotte-Pugh class A or B	Child-Turcotte-Pugh class C	p-value
Early mortality	30-day mortality	15 (17.6%)	1 (2.4%)	14 (31.8%)	<0.001
	90-day mortality	31 (36.5%)	7 (17.1%)	24 (54.5%)	<0.001
Late survival	Mean survival (days)	1474 ± 1743	1514 ± 1431	1422 ± 2114	0.315
	Interquartile range (days)	(137, 2215)	(459, 2035)	(67, 2459)	0.990

Data were presented by number (%) of patients or mean ± standard deviation.

### Predictors related with 30- or 90-day mortality: logistic regression analysis

In the stepwise multivariate analysis (Table 3), increased age (odds ratio, 1.102; p = 0.019), CTP class C (odds ratio, 18.707; p = 0.009), and bacteremia (odds ratio, 12.956; p = 0.025) at the time of PVO diagnosis were identified as predictors of 30-day mortality, and higher MELD score (odds ratio, 1.079; p = 0.003), presence of combined infection (odds ratio, 6.264; p = 0.003), and multiple spinal lesions (odds ratio, 3.838; p = 0.023) were identified as predictors of 90-day mortality.

**Table 3:** Predictors related with 30- or 90-day mortality: logistic regression analysis.

Variables	Category	30-day mortality						90-day mortality					
		Univariable			Multivariable (Stepwise)			Univariate			Multivariate (Stepwise)		
		odds ratio	95% confidence interval	p-value	odds ratio	95% confidence interval	p-value	odds ratio	95% confidence interval	p-value	odds ratio	95% confidence interval	p-value
Age		1.087	(1.016, 1.162)	0.015	1.102	(1.016, 1.196)	0.019						
Charlson comorbidity index													
Child-Turcotte-Pugh class	A and B	—	—	—	—	—	—	—	—	—	—	—	—
	C	18.667	(2.324, 149.903)	0.006	18.707	(2.053, 170.421)	0.009	5.829	(2.129, 15.954)	0.001			
Child-Turcotte-Pugh score		1.496	(1.140, 1.962)	0.004				1.380	(1.131, 1.685)	0.002			
MELD score		1.069	(1.017, 1.123)	0.009				1.084	(1.036, 1.135)	<0.001	1.079	(1.026, 1.135)	0.003
Hepatocellular carcinoma		4.000	(1.168, 13.698)	0.027									
Ascites													
Encephalopathy								3.632	(1.281, 10.296)	0.015			
GI bleeding		5.931	(1.729, 20.338)	0.005				5.390	(1.659, 17.508)	0.005			
Platelet (×10^3^/μL)								0.989	(0.980, 0.998)	0.016			
Total bilirubin (mg/dl)								1.140	(1.041, 1.248)	0.005			
Prothrombin time (INR)		1.903	(1.138, 3.182)	0.014				2.342	(1.437, 3.817)	0.001			
Serum creatinine (mg/dl)								1.402	(1.043, 1.885)	0.025			
Bacteremia		9.902	(1.232, 79.561)	0.031	12.956	(1.383, 121.346)	0.025	4.483	(1.498, 13.419)	0.007			
Presence of combined infection		6.500	(1.365, 30.954)	0.019				5.616	(1.982, 15.913)	0.001	6.264	(1.883, 20.842)	0.003
Other musculoskeletal infection		4.500	(1.193, 16.972)	0.026				6.955	(1.717, 28.174)	0.007			
Urinary tract infection								3.665	(1.394, 9.637)	0.009			
Multiple spinal lesion		3.750	(1.180, 11.913)	0.025				2.679	(1.056, 6.795)	0.038	3.838	(1.201, 12.263)	0.023

### Effect of early surgery on 30- or 90-day mortality: logistic regression analysis

Early surgery was performed in 34 patients (40.0%), of whom 9 (26.5%) underwent spinal instrumentation. The multivariate logistic regression analysis revealed that early surgical treatment was not associated with a statistically significant improvement in 30- or 90-day mortality (Table 4). A model (model 2 in Table 4) adjusted for all significant variables in the univariate analysis (Table 3) only showed a significantly lower odds ratio (0.005) for 30-day mortality in the patients who had an early surgery (p = 0.012).

**Table 4:** Effect of early surgery on 30-day or 90-day mortality: logistic regression analysis.

Categories of mortality	Method of adjustment		odds ratio	95% confidence interval	p-value
30-day mortality	Non-adjusted	None	0.707	(0.218, 2.287)	0.563
	Adjusted	Model 1	0.394	(0.083, 1.867)	0.241
		Model 2	0.002	(<0.001, 0.347)	0.018
90-day mortality	Non-adjusted	None	0.741	(0.298, 1.846)	0.520
	Adjusted	Model 3	0.588	(0.180, 1.922)	0.379
		Model 4	0.194	(0.026, 1.436)	0.108

Model 1: adjusted for age, Child-Turcotte-Pugh, and bacteremia.

Model 2: adjusted for age, Child-Turcotte-Pugh class, Child-Turcotte-Pugh score, MELD score, hepatocellular carcinoma, GI bleeding, prothrombin time (INR), sepsis, urinary tract infection, other musculoskeletal infection, and multiple spinal lesion.

Model 3: adjusted for MELD score, presence of combined infection, and multiple spinal lesion.

Model 4: adjusted for age, Child-Turcotte-Pugh class, Child-Turcotte-Pugh score, MELD score, hepatocellular carcinoma, GI bleeding, platelet, bilirubin, prothrombin time (INR), creatinine, sepsis, presence of combined infection, urinary tract infection, other musculoskeletal infection, and multiple spinal lesion.

### Treatment outcomes in patients with at least 90-day survival

Surgical treatment was performed in 51.9% (28 of 54 patients) of the survivors, and instrumentation was performed in 37% (20 of 54 patients) of the survivors (Table 5). The duration of antibiotic treatment and the length of hospital stay (from the PVO diagnosis) was longer in CTP C patients, however they were statistically insignificant (Table 5). Recurrence of PVO was identified in 11 patients (20.4%) and was more common in CTP C patients (p = 0.028) (Table 5).

**Table 5:** Treatment outcomes in patients with at least 90-day survival.

		All patients	Child-Turcotte-Pugh class A or B	Child-Turcotte-Pugh class C	p-value
Number of patients		54	34	20	
Presence of surgical treatment		28 (51.9)	19 (55.9)	9 (45.0)	0.440
Timing of initial surgery	Within 1 week	3 (5.6)	1 (2.9)	2 (10.0)	
	Between 1 and 4 weeks	20 (37.0)	14 (41.2)	6 (30.0)	
	After 4 weeks	5 (9.3)	4 (11.8)	1 (5.0)	
	None	24 (44.4)	15 (44.1)	11 (55.0)	
Presence of spinal instrumentation		20 (37.0)	14 (41.2)	6 (30.0)	0.411
Surgery related complication	Instrument failure	7 (13.0)	2 (5.9)	5 (25.0)	0.087
	Wound problem	5 (9.3)	4 (11.8)	3 (15.0)	0.347
Duration of antibiotics (days)		77.1 ± 28.3	71.2 ± 24.6	87.1 ± 31.9	0.065
Hospital stay (days)		80.4 ± 28.0	74.3 ± 24.3	90.6 ± 31.4	0.055
Recurrence		11 (20.4)	4 (11.8)	7 (35.0)	0.028

Data were presented by number (%) of patients or mean ± standard deviation.

## Discussion

As the first study to investigate the treatment outcome of PVO patients with cirrhosis, our study demonstrated that the 30- and 90-day mortality rates were 17.6% and 36.5%, respectively (Table 2). Multivariate analysis revealed increased age, CTP class C, and bacteremia at the time of PVO diagnosis as predictors of 30-day mortality, whereas higher MELD score, presence of combined infection, and multiple spinal lesions were predictors for 90-day mortality (Table 3). Early surgery did not lead to meaningful differences in the survival of the PVO patients with cirrhosis with respect to early mortality (Table 4).

Previous studies reported the early mortality of PVO patients, including in-hospital mortality or 90-day mortality ranging from 2.8% to 16.8%^27–32^. A recent study investigating the clinical outcome of PVO patients with hemodialysis reported an in-hospital mortality of 14.9% and 1-year mortality of 22.4%^15^. Compared with the results of previous studies, the mortality in our PVO patients with cirrhosis was considerably higher. The remarkable finding was the increased mortality observed between 30 and 90 days after PVO diagnosis (18.8%, 16/85 patients; Table 2), which was higher than the 30-day mortality (17.6%; Table 2). Although, inferring the cause of the higher mortality between 30 and 90 days after PVO diagnosis is beyond the scope of our study, we could explain the cause as follows: first, PVO patients generally require long-term hospitalization for the administration of intravenous antibiotics for at least 6 weeks^8^, which paradoxically increases the risk of hospital-acquired infections such as *Clostridium difficile* infection or the risk of recurrence by multidrug-resistant organisms. In addition, long-term intravenous antibiotics can attenuate liver or kidney function, which negatively influences the survival of patients. Second, pain and disability from the spinal structural instability negatively influences survival. During PVO treatment, significant bone loss occurs directly by causative organisms and indirectly by disuse-type bone loss^33–35^. Such bone loss can induce structural instability, which leads to neurological deficit, spinal deformity, and even death^36,37^. Therefore, permanent, and extensive stabilization using spinal instrumentation is often required after debridement or neural decompression. However, such long instrumentation is technically demanding in patients with cirrhosis, and it even fails in such patients with osteoporosis and progressive bone loss^38^.

The factors related with liver function were consistently significant predictors of PVO patients’ survival (Table 3) (Fig. 2), and these results are in line with the results of other types of infection in patients with cirrhosis^39,40^. The multivariate analysis identified CTP class and MELD score as significant predictors of 30-day and 90-day mortality, respectively (Table 3). Within 30 days after PVO diagnosis, only one of the patients with CTP class A or B died (2.4%, Table 2). However, one third of the patients with CTP class C died within 30 days (31.8%, Table 2). The Charlson comorbidity index score did not show a significant association with 30-day (p = 0.125; odds ratio, 1.138), 90-day mortality (p = 0.258; odds ratio, 1.083), and late mortality (p = 0.931, odds ratio, 0.994).

In addition to liver function, the significant predictor of mortality in PVO patients with cirrhosis was the gross extent of infection indicated by the presence of multiple spinal lesions and combined infection (Table 3). The diagnosis of PVO is frequently delayed in clinical practice^41^. Unfortunately, such delayed diagnosis of PVO is believed to cause extensive musculoskeletal involvement of the spine and neurological and structural instabilities in patients with cirrhosis. Approximately one-third of the cohort (34.1%; Table 1) had multiple spinal lesions, and two-thirds of the cohort (61.2%; Table 2) had extensive spinal involvement beyond 3 vertebral bodies. According to the classification of Pola *et al*., 94.1% of the cohort (80/85 patients) had structural instability (type B) or neurological compromise (type C), which theoretically requires surgical treatment^18^. Compared with the results of previous reports^15,42^, our results showed that PVO patients with cirrhosis are considered to have an even more extensive spinal involvement than other groups of PVO patients. We hypothesized that immune dysfunction^11^ and impaired bony microarchitecture^14^ contributes to aggressive infection.

Combined bacterial infection is common in patients with cirrhosis^7^ and reported to be closely related to high short-term mortality^7,43^. Therefore, combined infection should be considered in studies about infection-related treatment outcome in patients with cirrhosis. In our study, combined infection presented in more than half of the patients (56.5%; Table 1), and the most common combined infection was urinary tract infection (30.6%; Table 1). The multivariate analysis confirmed that combined infection is closely related with the mortality of PVO patients with cirrhosis (Table 3). In this respect, clinicians should pay great attention to the presence of combined infection in PVO patients with cirrhosis. If PVO patients with cirrhosis are considered to have combined infection in other organs or if patients with cirrhosis are receiving treatment for infection in other organs show symptoms or signs of PVO, clinicians should be aware that such a combined infection is closely related to patient survival.

The establishment of prognostic factors related to mortality should be connected to early treatment strategies. In our study, aged patients with advanced cirrhosis who had combined infection or multiple spinal lesions were identified to have high mortality rates. Therefore, this group of patients should be targeted for an aggressive diagnostic approach using spinal MRI and intensive monitoring and treatment strategies. If the diagnosis is established, broad-spectrum antibiotics should be administered as early as possible; this is a prerequisite to decrease the burden of infection and to prevent early mortality in PVO patients with cirrhosis. In this respect, early surgical drainage can be theoretically suggested as a possible treatment option to rapidly remove epidural and intraosseous abscesses, which occur in relatively avascular areas where antibiotics cannot easily reach and require long-term intravenous antibiotic administration^8^. However, early surgical treatment did not show a statistically significant outcome in our study (Table 4). The survival of the PVO patients with cirrhosis was strongly influenced by their liver function (Table 1), and early surgical treatment was believed to be insufficient for a clinically significant decrease in infection burden in these patients with such wide extent of combined or multiple spine infection (Table 1). However, a multivariate analysis revealed a significantly lower odds ratio for 30-day mortality in patients with early surgery (odds ratio, 0.002, p = 0.018, model 2 in Table 4). A large-scale multicenter study is required to confirm the effect of early surgery on the survival of PVO patients with cirrhosis.

The main limitation of our study is its retrospective design, and some unidentified confounders may have influenced the clinical outcomes of our patients. Precise clinical factors including the method of antibiotic treatment, method of surgical treatment including spinal instrumentation, surgery-related complications may have influenced the treatment outcomes, especially mortality, of our cohort. However, owing to the high early mortality in our cohort and small sample size, inclusion of such various clinical factors to estimate their association with clinical outcome was difficult. Next, due to limited population of our cohorts, we only investigated association between the presence of combined infection and the mortality of PVO patients. Further large-sized studies are required to investigate the individual impact of each type of combined infection on the mortality of PVO patients with cirrhosis.

In conclusion, the 30- and 90-day mortality rates of the PVO patients with cirrhosis were 17.6% and 36.5%, respectively. Attention should be paid to the high mortality between 30 and 90 days after PVO diagnosis (18.9%), which was higher than the 30-day mortality. Liver function was consistently a strong predictor of mortality in PVO patients with cirrhosis. We also identified increased age and bacteremia at the time of PVO diagnosis as predictors of 30-day mortality; and the presence of combined infection, and multiple spinal lesions as predictors of 90-day mortality. This group of patients should be targeted for an aggressive diagnostic approach using spinal MRI and intensive monitoring and treatment strategies.
